# Base-modified UDP-sugars reduce cell surface levels of P-selectin glycoprotein 1 (PSGL-1) on IL-1β-stimulated human monocytes

**DOI:** 10.1093/glycob/cww053

**Published:** 2016-10-18

**Authors:** Varsha Kanabar, Lauren Tedaldi, Jingqian Jiang, Xiaodan Nie, Irina Panina, Karine Descroix, Francis Man, Simon C Pitchford, Clive P Page, Gerd K Wagner

**Affiliations:** 2 Sackler Institute of Pulmonary Pharmacology; 3 Institute of Pharmaceutical Science, King's College London, Franklin Wilkins Building, London SE1 9NH, UK; 4 Department of Chemistry, Faculty of Natural & Mathematical Sciences, King's College London, Britannia House, 7 Trinity Street, London, SE1 1DB, UK; 5 School of Pharmacy, University of East Anglia, Norwich, NR4 7TJ, UK

**Keywords:** glycosyltransferase, inhibitor, interleukin-1β, monocytes, P-selectin glycoprotein-1

## Abstract

P-selectin glycoprotein ligand-1 (PSGL-1, CD162) is a cell-surface glycoprotein that is expressed, either constitutively or inducibly, on all myeloid and lymphoid cell lineages. PSGL-1 is implicated in cell–cell interactions between platelets, leukocytes and endothelial cells, and a key mediator of inflammatory cell recruitment and transmigration into tissues. Here, we have investigated the effects of the β-1,4-galactosyltransferase inhibitor 5-(5-formylthien-2-yl) UDP-Gal (5-FT UDP-Gal, compound **1**) and two close derivatives on the cell surface levels of PSGL-1 on human peripheral blood mononuclear cells (hPBMCs). PSGL-1 levels were studied both under basal conditions, and upon stimulation of hPBMCs with interleukin-1β (IL-1β). Between 1 and 24 hours after IL-1β stimulation, we observed initial PSGL-1 shedding, followed by an increase in PSGL-1 levels on the cell surface, with a maximal window between IL-1β-induced and basal levels after 72 h. All three inhibitors reduce PSGL-1 levels on IL-1β-stimulated cells in a concentration-dependent manner, but show no such effect in resting cells. Compound **1** also affects the cell surface levels of adhesion molecule CD11b in IL-1β-stimulated hPBMCs, but not of glycoproteins CD14 and CCR2. This activity profile may be linked to the inhibition of global Sialyl Lewis presentation on hPBMCs by compound **1**, which we have also observed. Although this mechanistic explanation remains hypothetical at present, our results show, for the first time, that small molecules can discriminate between IL-1β-induced and basal levels of cell surface PSGL-1. These findings open new avenues for intervention with PSGL-1 presentation on the cell surface of primed hPBMCs and may have implications for anti-inflammatory drug development.

## Introduction

P-selectin glycoprotein ligand-1 (PSGL-1) is a cell-surface glycoprotein that is expressed, either constitutively or inducibly, on all myeloid and lymphoid cell lineages (Laszik et al. 1996). PSGL-1 binds to P-, E- and L-selectin under flow conditions, and is the main physiological selectin ligand for leukocyte rolling on activated endothelial cells (Carlow et al. 2009; Zarbock et al. 2011). In addition to mediating leukocyte tethering and rolling, PSGL-1 also transduces signals into rolling leukocytes, and triggers platelet/leukocyte aggregate formation and other forms of platelet/leukocyte interaction (Carlow et al. 2009; Sreeramkumar et al. 2014). It has previously been shown that platelets are essential for pulmonary leukocyte recruitment, bronchial hyperresponsiveness and airway remodeling in animals induced by both allergic and non-allergic stimuli (Pitchford et al. 2003, 2004, 2005; Zarbock et al. 2006; Tamagawa-Mineoka et al. 2007; Pitchford et al. 2008; Kornerup et al. 2010). These events are driven by the cell surface levels of PSGL-1 on leukocytes, and of P-selectin on the surface of activated circulating platelets, as evidenced by the fact that administration of either anti-PSGL-1 or anti-P-selectin antibodies almost entirely abrogates pulmonary recruitment of eosinophils, neutrophils and T cells in mice (Pitchford et al. 2005, 2008; Kornerup et al. 2010). As a critical step in the cascade of events required for the recruitment of inflammatory cells, the P-selectin/PSGL-1 interaction therefore represents a promising target for therapeutic intervention, e.g., in chronic inflammatory conditions such as asthma and COPD (Binder and Ernst 2011).

**Fig. 1.**
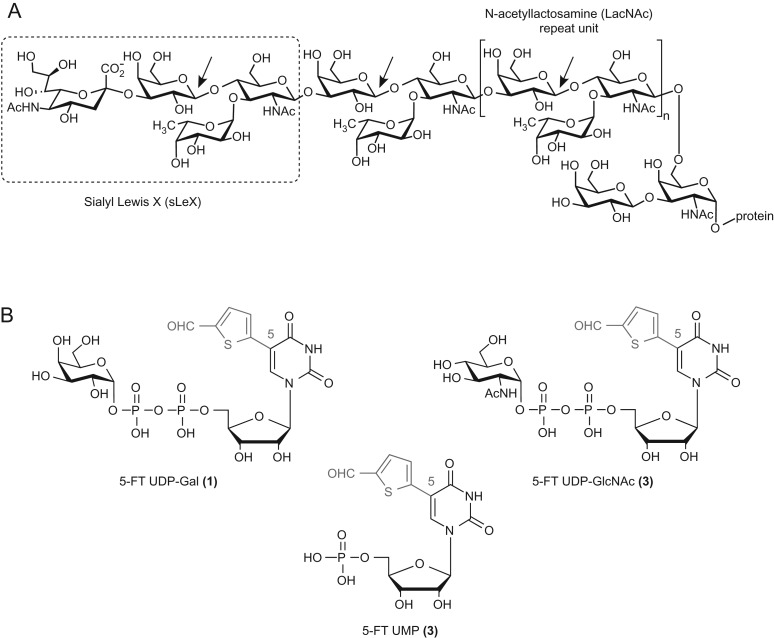
(**A**) Structure of the PSGL-1 glycan epitope, including the terminal sLeX tetrasaccharide, and the LacNAc repeat unit connecting it to protein. Arrows indicate the linkages generated by the galactosyltransferase β-1,4-GalT. (**B**) Chemical structures of inhibitors **1–3**. The synthetic 5-(5-formylthien-2-yl) substituent is shown in red. This figure is available in black and white in print and in color at *Glycobiology* online.

There are two main pharmacological anti-selectin strategies: the direct disruption of the selectin/selectin-ligand interaction with a selectin antagonist (Binder and Ernst 2011), and the reduction of cell surface levels of functional PSGL-1, the main selectin ligand. Structurally, PSGL-1 is a homodimer composed of two heavily *O*-glycosylated 120-kDa monomers (Wilkins et al. 1996, reviewed in Moore 1998). The first 19 amino acids of PSGL-1, which are sufficient for high-affinity binding of PSGL-1 to P-selectin, include both a tyrosine sulfation motif (Sako et al. 1995) and an *O*-glycosylation site at Threonine-16 (Goetz et al. 1997). The tetrasaccharide Sialyl Lewis X (sLeX, Fig. 1) is present as a terminal epitope on a significant proportion of *O*-glycans in this position, and is essential for selectin-binding of PSGL-1 (Wilkins et al. 1996; Somers et al. 2000). Several glycomimetics, such as GMI-1070 (Egger et al. 2013; Wun et al. 2014; Telen et al. 2015) and bimosiamose (Beeh et al. 2006; Kirsten et al. 2011), have been clinically successful as pan-selectin antagonists in a range of therapeutic areas (Ernst and Magnani 2009). Inhaled bimosiamose has shown modest clinical efficacy in subjects with allergen-induced asthma following allergen challenge (Beeh et al. 2006), as well as in a Phase-II trial in patients with COPD (Kirsten et al. 2011). More recently, GMI-1070 was shown to successfully reduce vaso-occlusive events in a Phase-II study of patients with sickle cell disease (Telen et al. 2015). While these results have provided clinical proof-of-concept for the therapeutic value of an anti-selectin strategy, chemical pan-selectin antagonists tend to be relatively large molecules, due to the shallow nature of the P-selectin ligand-binding site (Somers et al. 2000). This can lead to poor pharmacokinetics and very likely precludes p.o. administration. Bimosiamose, for example, has a molecular weight of 867, and requires mandatory administration by inhalation.

An alternative approach to the direct disruption of the P-selectin/PSGL-1 interaction with selectin antagonists is the reduction of cell surface levels of functional PSGL-1. This can be achieved, e.g., by inhibiting the biosynthesis of its functionally relevant glycans, in particular sLeX. In unstimulated cells, inhibition of sLeX biosynthesis has been accomplished with metabolic inhibitors and substrate decoys for glycosyltransferases that affect galactosylation (Sarkar et al. 1995; Brown et al. 2009), sialylation (Rillahan et al. 2012), fucosylation (Rillahan et al. 2012; Zandberg et al. 2012; Belcher et al. 2015) or incorporation of *N*-acetylglucosamine (Marathe et al. 2010; Barthel et al. 2011). Reducing the cell surface levels of functional PSGL-1 offers at least three important advantages over the direct disruption of the P-selectin/PSGL-1 interaction: firstly, reduced levels of cell surface PSGL-1 will affect adhesion processes mediated by all selectins, thus overcoming potential operational redundancy between individual selectins. Secondly, the levels of PSGL-1 on the cell surface can be reduced by inhibition of PSGL-1 biosynthetic enzymes. This can be achieved with classical, drug-like small molecules that are orally bioavailable, in contrast to selectin antagonists. Thirdly, and perhaps most importantly, as the expression of PSGL-1 is upregulated during inflammation (Carlow et al. 2009), a PSGL-1 biosynthesis inhibitor may allow the discrimination between PSGL-1 expression under physiological and pro-inflammatory conditions. Such a discrimination, which is not possible with a selectin antagonist, may selectively reduce the pro-inflammatory effects of PSGL-1, but have only a limited effect on the constitutive cell surface levels of PSGL-1 that are required for normal host defense, resulting in a favorable safety profile.

Previous studies with metabolic inhibitors and substrate decoys have all been concerned with basal PSGL-1 and/or sLeX expression in unstimulated cells (Sarkar et al. 1995; Brown et al. 2009; Marathe et al. 2010; Barthel et al. 2011; Rillahan et al. 2012; Zandberg et al. 2012; Belcher et al. 2015). Whether a small molecular weight inhibitor may be able to discriminate between the cell surface presentation of PSGL-1 under basal and pro-inflammatory conditions has, to the best of our knowledge, not been investigated to date. It was the main goal of the present study to explore this exciting possibility. We have established a flow cytometry protocol to study the cell surface levels of PSGL-1 on CD14+ human peripheral blood mononuclear cells (hPBMCs) from healthy donors, both in the absence and presence of a pro-inflammatory stimulus (IL-1β). We have used this assay to investigate the effects of a new class of glycosyltransferase (GT) inhibitors, exemplified by compound **1** (Fig. 1), on the levels of cell surface PSGL-1 in basal and IL-1β-induced hPBMCs. Compound **1** is a derivative of the natural galactosyltransferase (GalT) donor substrate UDP-galactose (UDP-Gal) and a broad-spectrum GalT inhibitor (Pesnot et al. 2010; Descroix et al. 2012; Jørgensen et al. 2013; Tedaldi et al. 2014). GalT inhibitor **1** and the related compounds **2** and **3** were chosen for this study, as the biosynthesis of functional, sLeX-decorated PSGL-1 requires several GTs, including β-1,4-GalT (Fig. 1). β-1,4-GalT catalyzes the transfer of d-galactose from a UDP-Gal donor to a terminal *N*-acetyl-d-glucosamine (GlcNAc) residue on the acceptor (Sperandio 2006). The enzyme is required for the biosynthesis of not only the sLeX epitope on PSGL-1, but also the LacNAc linker, which connects sLeX to the protein backbone (Fig. 1). β-1,4-GalT is a particularly interesting target for the selective intervention with PSGL-1 levels under pro-inflammatory conditions, as its expression is upregulated in inflammation (Qian et al. 2007). All three compounds reduce the cell surface presentation of PSGL-1 in a concentration-dependent manner in IL-1β-stimulated cells, but not in resting cells. Additional experiments, including selectivity, cell uptake and stability experiments, suggest that these compounds may have an intracellular mode of action, and are probably acting on sLeX biosynthesis. This is the first report on the cellular activity of this new class of GT inhibitors. Our findings open new avenues for intervention with cell surface levels of PSGL-1 and subsequent function, and may have direct implications for novel anti-inflammatory drug development.

## Results

### Time course of cell surface PSGL-1 levels on hPBMCs

It is known that, following stimulation of hPBMCs with a range of pro-inflammatory stimuli, PSGL-1 undergoes a time-dependent process of shedding and re-synthesis (Davenpeck et al. 2000; Marsik et al. 2004). In order to identify the optimal time point for inhibitor studies, we first examined the levels of PSGL-1 on the cell surface of CD14 positive (CD14+) hPBMCs at 0, 1, 24 and 48 h. We studied cell surface PSGL-1 levels both on unstimulated hPBMCs (constitutive PSGL-1 expression), and on hPBMCs stimulated with the pro-inflammatory cytokine IL-1β at either 10 or 100 ng/mL. Cell surface PSGL-1 was detected by flow cytometry with the monoclonal mouse anti-hPSGL-1-PE-conjugated antibody CD162 (clone KPL-1). CD162/KPL-1 recognizes the N-terminus of PSGL-1 (Snapp et al. 1998), although there is some uncertainty regarding the exact epitope. It has been suggested that the CD162/KPL-1 epitope comprises amino acid residues 5–11 (YEYLDYD), including a consensus tyrosine sulfation motif (Snapp et al. 1998), but not the sLeX glycan (Marathe et al. 2010), despite the close proximity of residues 5–11 to the *O*-glycosylation site at Threonine-16. To quantify the changes in cell surface PSGL-1 levels, we determined both the population of CD14+ cells displaying PSGL-1 (in % of the total population) and the average cell surface levels of PSGL-1 per cell (as indicated by mean fluorescence intensity, MFI).

**Fig. 2.**
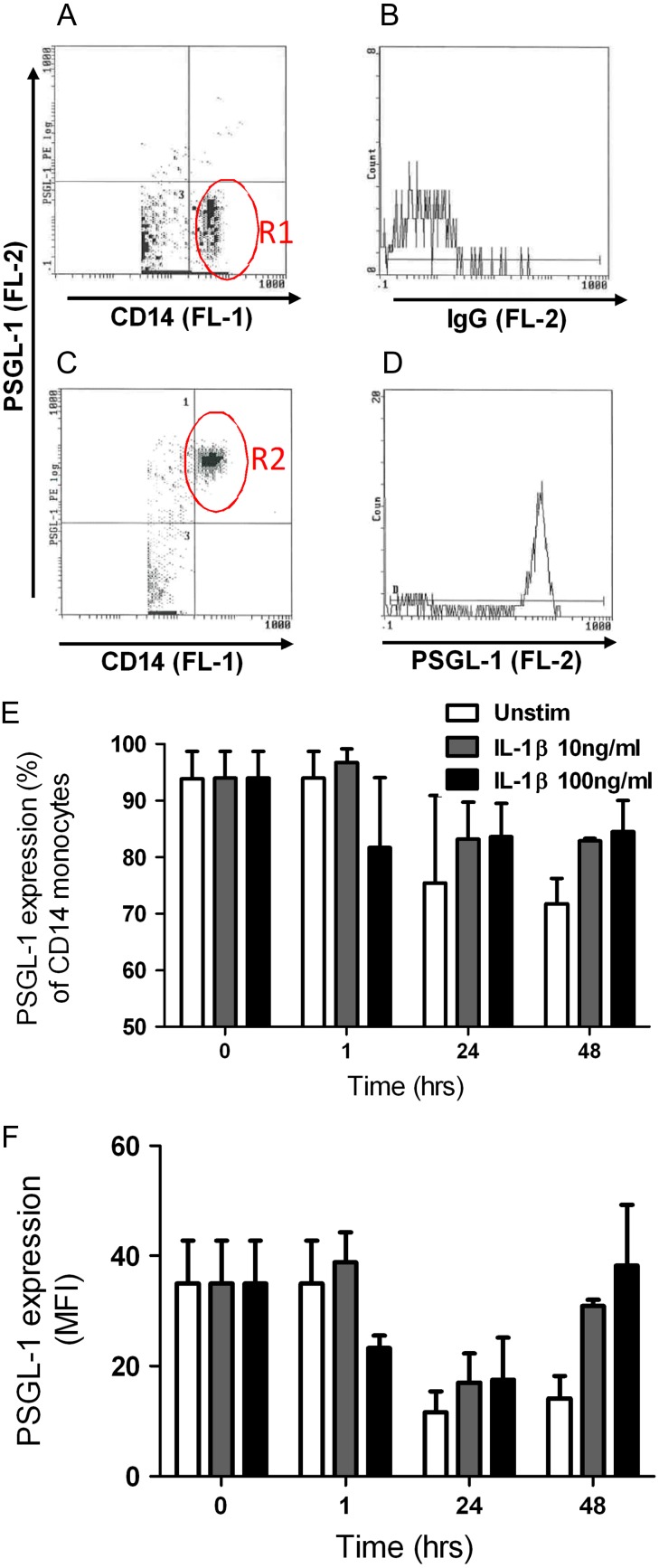
Characterization of IL-1β-induced PSGL-1 cell surface levels on human monocytes. (**A**) Human mononuclear cells were isolated from healthy donors (*n* = 2) and incubated with anti-CD14-FITC to identify the monocyte population by flow cytometry (region R1). (**B**) CD14+ monocytes incubated with isotype control IgG-PE to reveal background fluorescence. (**C**) Scatter graph revealing the percentage of resting CD14+ monocytes displaying PSGL-1 (region R2). (**D**) Representative histogram of CD14+ monocytes and mean fluorescence intensity of PSGL-1 levels (anti-PSGL-1-PE). hPBMCs were either treated with media alone (white bar), 10 ng/mL recombinant human IL-1β (gray bar), or 100 ng/mL IL-1β (black bar) for 0–48 hr. Cells were harvested and co-stained with PE CD162, FITC CD14 and isotype matched controls. (**E**) Percentage of CD14+ monocytes displaying PSGL-1. (**F**) Mean fluorescent intensity (MFI) of cell surface PSGL-1 levels on monocytes. Data represent mean ± SEM. This figure is available in black and white in print and in color at *Glycobiology* online.

At 0 h, almost the entire population of CD14+ hPBMCs (93.9 ± 4.9%, *n* = 2) displayed cell surface PSGL-1 constitutively (Fig. 2 and Table I). Over the 48-h incubation period, the size of this PSGL-1-presenting population fluctuated, both in unstimulated and IL-1β-treated cells (Fig. 2E). A maximal reduction to 71.8 ± 4.5% was observed for CD14+ cells presenting PSGL-1 constitutively at the 48 h timepoint (Table I). MFI readings showed a clear time-dependent decrease of constitutive PSGL-1 levels on CD14+ cells after both 24 h and 48 h incubation, by 62.5 ± 19.3% and 60.3 ± 2.8%, respectively (Fig. 2F and Table I). At 48 h, IL-1β-stimulated hPBMCs appeared to recover the levels of cell surface PSGL-1 back to those observed at 0 h, whereas unstimulated hPBMCs had no such recovery (Fig. 2F and Table I). Thus, cell surface levels of PSGL-1 on hPBMCs treated with IL-1β exceeded those on unstimulated cells by approximately 2.5-fold (10 ng/mL IL-1β: 2.4 ± 0.6 fold; 100 ng/mL IL-1β: 2.7 ± 0.02 fold; Fig. 2F and Table I).

**Table I.** Changes in cell surface levels of PSGL-1 over time

Time (h)	Treatment			PSGL-1 levels
	Unstimulated	IL-1β 10 ng/mL	IL-1β 100 ng/mL	
0	93.9 ± 4.9	–	–	%
	35 ± 7.9	–	–	MFI
24	75.5 ± 15.5	83.1 ± 6.6	83.7 ± 5.9	%
	11.6 ± 3.8	17.0 ± 5.3	17.5 ± 7.6	MFI
48	71.8 ± 0.5	82.9 ± 0.5	84.5 ± 5.5	%
	14.1 ± 4.1	30.9 ± 1.1	38.3 ± 10.9	MFI

**Fig. 3.**
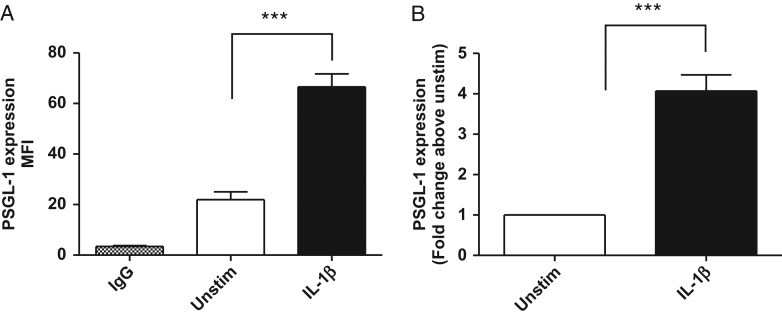
IL-1β-induced PSGL-1 cell surface levels on human monocytes. Human monocytes were isolated from healthy donors (*n* = 17) and incubated in the presence (black bar) and absence (white bar) of 10 ng/mL recombinant human IL-1β for 72 h. Cells were harvested and co-stained with PE CD162, FITC CD14 and isotype matched controls. (**A**) Represents mean fluorescent intensity (MFI) values in unstimulated and IL-1β-treated (10 ng/mL) cells and (**B**) fold change in PSGL-1 levels upon stimulation with IL-1β, relative to unstimulated (media alone) control values. IL-1β significantly induced a 4.0 ± 0.4 fold increase in PSGL-1 cell surface levels (***P < 0.0001, students paired *t* test, *n* = 21). Data represent mean ± SEM from 21 independent experiments using monocytes isolated from 17 human donors (10M/7F).

Despite the restricted number of hPBMC donors, these initial results suggested strongly that longer incubation times increase the window between constitutive and IL-1β-induced PSGL-1 cell surface levels. To maximize cell surface levels of PSGL-1 after shedding and recovery, we therefore extended this preliminary time course to 72 h post IL-1β stimulation. We found that 10 ng/mL IL-1β significantly increased cell surface levels of PSGL-1 by approximately 4-fold (4.1 ± 0.4) compared to unstimulated CD14+ hPBMCs after 72 h incubation (*P* < 0.0001, Fig. 3). The MFI was 22.0 ± 3.1 in unstimulated hPBMCs, and 66.6 ± 5.2 in IL-1β-stimulated cells. These conditions therefore provided an appropriate model and timeframe to investigate inhibitors of IL-1β-induced PSGL-1 levels on the cell surface of hPBMCs.

### Effects of compound **1** on the cell surface levels of PSGL-1 on hPBMCs

**Fig. 4.**
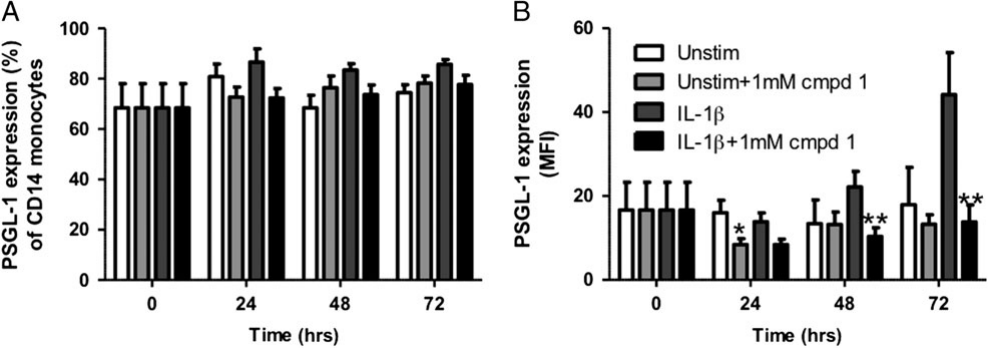
Effect of compound **1** (1 mM) on PSGL-1 cell surface levels. Monocytes from whole blood were incubated for 0, 24, 48 and 72 hours with or without IL-1β (10 ng/mL), and with or without compound **1**. Samples were analyzed using flow cytometry. Graphs show (**A**) the percentage of mononuclear cells displaying cell surface PSGL-1, (**B**) the mean fluorescence intensity (MFI) of PSGL-1 cell surface levels. Data are expressed as mean ± SEM (*n* = 4–6). *P < 0.01 and **P < 0.05 between IL-1β-stimulated groups.

Next, we investigated the effect of compound **1**, at a single concentration, on the cell surface levels of PSGL-1. hPBMCs were incubated with 1 mM compound **1** for 1 h, before cells were treated with either media alone or 10 ng/mL IL-1β and further incubated for 0, 24, 48 or 72 hours. There was no significant inhibitory effect of compound **1** on the percentage of monocytes displaying PSGL-1 (Fig. 4A). MFI readings showed a maximal IL-1β-induced increase in PSGL-1 levels, by 2.5-fold, at 72 h compared to unstimulated levels (Fig. 4B, Table II). In the presence of compound **1,** this response was significantly decreased by 74.7 ± 6.8%, back to the basal levels observed at 0 h. Compound **1** also inhibited IL-1β-induced cell surface PSGL-1 presentation by 40% and 53% at 24 h and 48 h, respectively. These data demonstrate that 1 mM of compound **1** reduces IL-1β-induced levels of PSGL-1 on the cell surface of CD14+ hPBMCs at all time points examined. In contrast, the MFI of PSGL-1 levels on resting hPBMCs was not significantly different in the presence or absence of compound **1**, with the exception of a small, albeit significant, decrease at 24 h (8.4 ± 1.4 vs. 16.0 ± 2.9, *P* < 0.05) (Fig. 4B).

**Table II.** IL-1β-induced presentation of cell surface PSGL-1 in the presence and absence of compound **1**

Time (h)	Treatment		PSGL-1 levels
	IL-1β	IL-1β + cmpd **1**	
0	16.7 ± 6.6	16.7 ± 6.6	MFI
24	13.9 ± 2.1	8.4 ± 1.3	MFI
48	22.2 ± 3.7	10.4 ± 2.1	MFI
72	44.2 ± 10.0	13.8 ± 4.1	MFI

### Effects of compound **1** on the cell surface levels of sLeX on hPBMCs

**Fig. 5.**
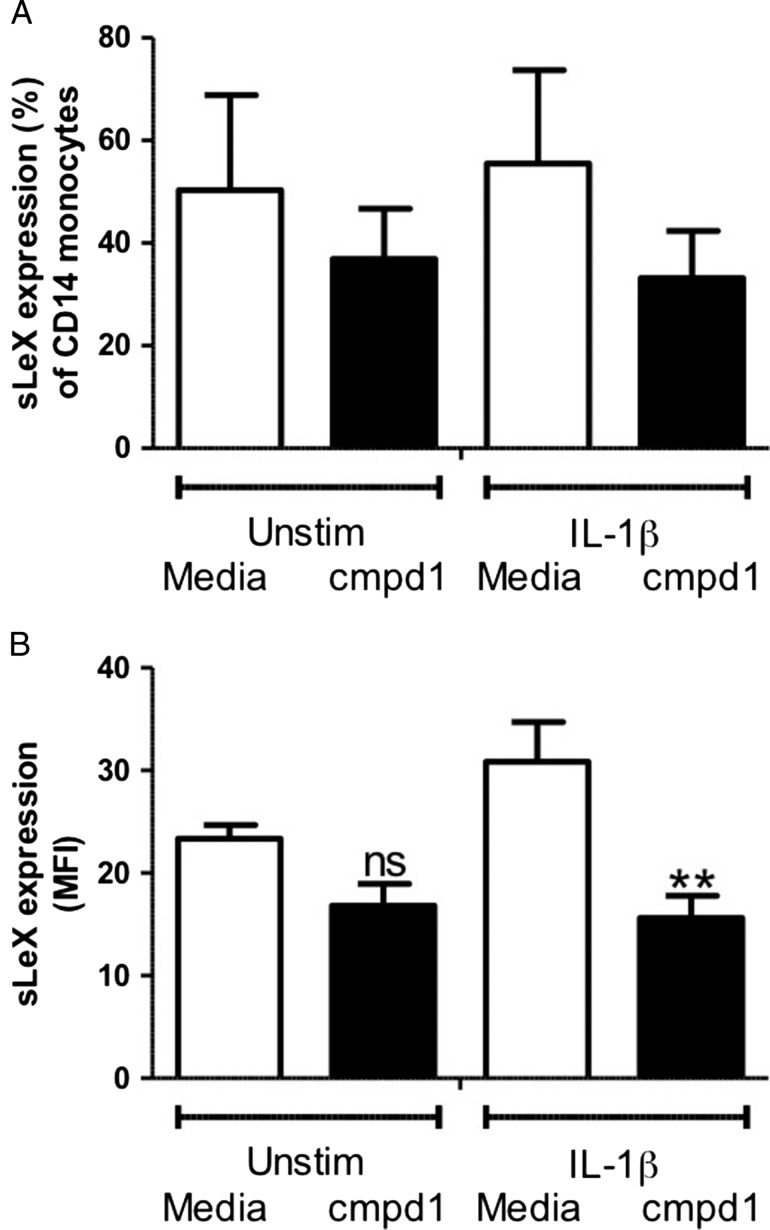
Effect of compound **1** (1 mM) on sLeX cell surface levels. Monocytes from whole blood were incubated for 72 hours with or without IL-1β (10 ng/mL), and with or without compound **1**. Samples were analyzed using flow cytometry. Graphs show (**A**) the percentage of CD14+ cells displaying cell surface sLeX; (**B**) the mean fluorescence intensity (MFI) of sLeX levels. Data are expressed as mean ± SEM (*n* = 4–5). **P < 0.01 between IL-1β-stimulated groups. ns indicates not significant.

In vitro, compound **1** is a potent, broad-spectrum inhibitor of GalTs (Pesnot et al. 2010; Descroix et al. 2012; Tedaldi et al. 2014), including β-1,4-GalT, which is required for sLeX biosynthesis (Fig. 1). To assess the effect of compound **1** on global sLeX presentation on hPBMCs, we repeated our inhibition experiments using an antihuman-sLeX (CD15s) antibody. Incubation with compound **1** did not affect the percentage of CD14+ hPBMCs displaying sLeX on either IL-1β-stimulated cells or controls (Fig. 5A). However, after 72 h incubation, a significant decrease in cell surface levels of sLeX (MFI) was observed for hPBMCs treated with both IL-1β and compound **1** (1 mM), compared to cells treated with IL-1β only (*P* < 0.01, Fig. 5B). This suggests that, as expected for a GalT inhibitor, compound **1** reduces global sLeX presentation on hPBMCs.

### Epitope specificity of the anti-sLeX and anti-PSGL-1 monoclonal antibodies

**Fig. 6.**
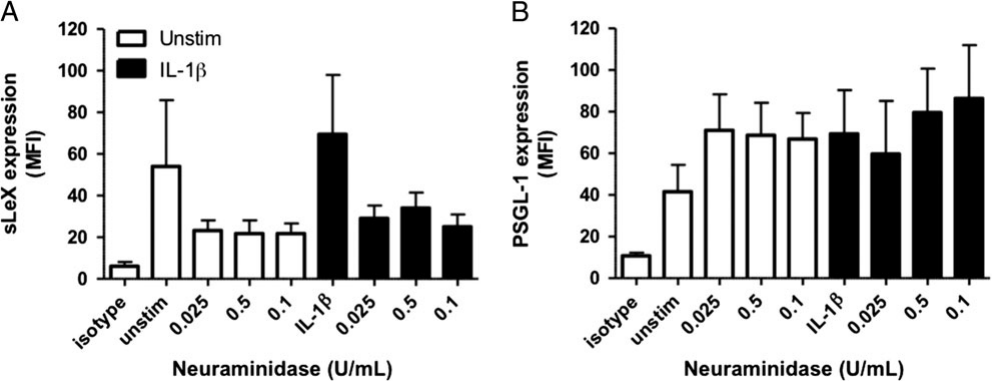
Neuraminidase treatment. Monocytes from whole blood were incubated for 72 hours with or without IL-1β (10 ng/mL). Samples were treated with or without neuraminidase (0.025–0.1 U/mL) and analyzed using flow cytometry. Graphs show (**A**) the MFI of sLeX cell surface levels, and (**B**) the MFI of PSGL-1 cell surface levels. Data are expressed as mean ± SEM (*n* = 4–6).

To confirm the epitope specificity of the anti-sLeX monoclonal antibody for the α-2,3-sialosylated form of lacto-*N*-fucopentaose III (i.e. sLeX), we removed cell surface sialic acid by treating hPBMCs with neuraminidase prior to addition of antibody. As expected, neuraminidase (0.025–0.1 U/mL) treatment prevented sLeX antibody recognition (Fig. 6A). In contrast, it had no significant effect on the ability of the anti-human PSGL-1 antibody CD162/KPL-1 to recognize its epitope in the same cells, whether stimulated with IL-1β or not (Fig. 6B). This suggests that the CD162/KPL-1 epitope does not include sLeX. This result is in keeping with the findings of Marathe and coworkers (Marathe et al. 2010) that neuramidinase treatment does not affect the detection of PSGL-1 levels on HL-60 cells by the same CD162/KPL-1 antibody used in our study.

### Effects of compound **1** on the cell surface levels of CD14, CD11b and CCR2 on hPBMCs

**Fig. 7.**
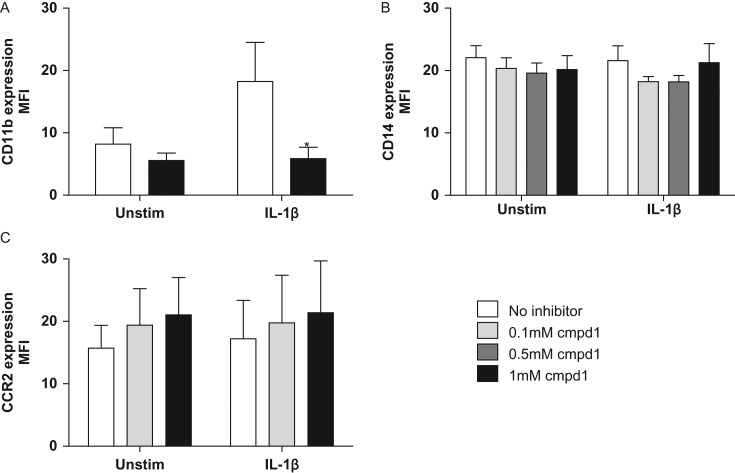
Effect of compound **1** on cell surface levels of CD11b, CD14 and CCR2 on untreated (“unstim”) and IL-1β-treated (“IL-1β”) monocytes. Monocytes from whole blood were incubated for 72 hours with or without IL-1β (10 ng/mL), in the presence or absence of compound **1**. Samples were analyzed using flow cytometry. Graphs show (**A**) the MFI of CD14+ cells displaying CD11b, (**B**) the MFI of CD14 cell surface levels, and (**C**) the MFI of CCR2 cell surface levels. Data are expressed as mean ± SEM (*n* = 4–9). *P < 0.01 compared to IL-1β-stimulated groups.

In order to explore its target selectivity, we investigated the effect of compound **1** on the levels of three cell surface proteins other than PSGL-1: CD11b, our monocyte marker CD14, and the chemokine receptor CCR2, a G-protein-coupled receptor for monocyte chemoattractant protein-1. Like PSGL-1, CD11b is decorated with sLeX moieties (Zen et al. 2007). 10 ng/mL IL-1β induced a 2.2-fold increase in cell surface CD11b levels after 72 h incubation (Fig. 7A). This response was significantly inhibited by 1 mM compound **1** back to constitutive levels (Fig. 7A, *P* < 0.05, *n* = 10). In contrast to CD11b, cell surface levels of CD14 and CCR2 were not increased by IL-1β-treatment (Fig. 7B & C). Moreover, compound **1** had no effect upon cell surface levels of either CD14 (Fig. 7B, *n* = 11, 72 h), or CCR2 (Fig. 7C, *n* = 4, 24 h), neither in the presence nor absence of IL-1β. Collectively, these data suggest that compound **1** inhibits pathways associated with the cell surface presentation of PSGL-1, sLeX and CD11b, but not of CD14 and CCR2.

### Concentration-dependent reduction of cell surface levels of PSGL-1 by compounds **1–3**

**Fig. 8.**
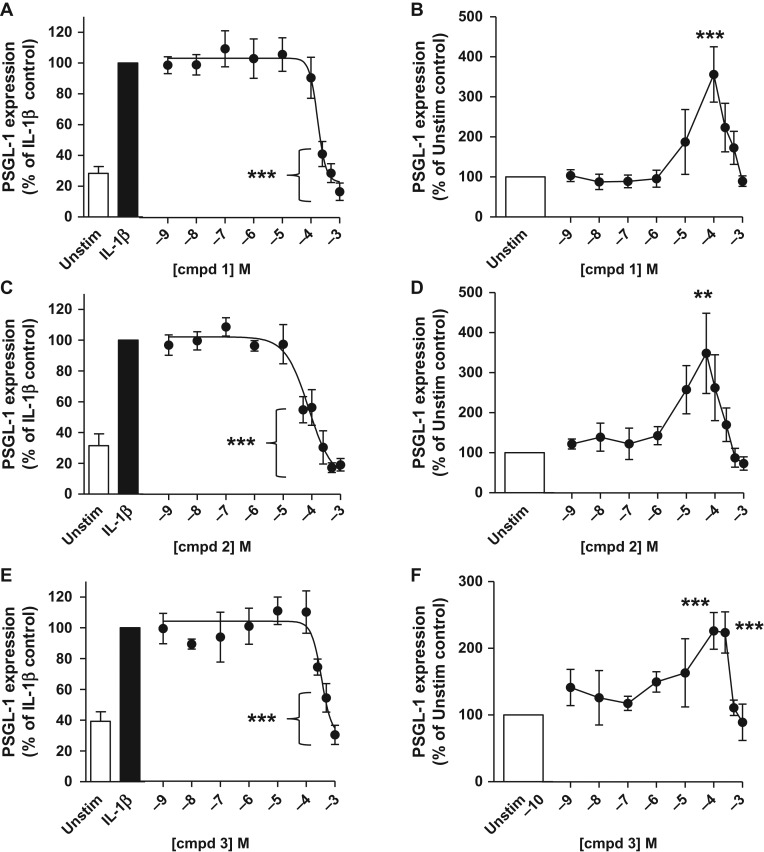
Compounds **1** (panels **A** & **B**), **2** (panels **C** & **D**) and **3** (panels **E & F**) inhibit IL-1β-induced PSGL-1 cell surface levels on human monocytes, and potentiate basal PSGL-1 presentation. Human monocytes were isolated from healthy donors (**1**: *n* = 9; **2**: *n* = 7; **3**: *n* = 9) and incubated in the presence (black bar) or absence (white bar) of 10 ng/mL recombinant human IL-1β for 72 h. Cells were harvested and co-stained with PE CD162, FITC CD14 and isotype-matched controls. Concentration-response curves in panels **A**, **C** and **E** show PSGL-1 levels in %, relative to maximal PSGL-1 levels (***P < 0.0001, one-way ANOVA; **1**: *n* = 9; **2**: *n* = 7; **3**: *n* = 9), upon stimulation with 10 ng/mL IL-1β and incubation with inhibitor for 72 h (**A**: **1**; **C**: **2**; **E**: **3**). IC_50_ values extracted from concentration-response curves in panels **A**, **C** and **E** are 174 µM (for compound **1**), 77 µM (for compound **2**) and 343 µM (for compound **3**). Concentration-response curves in panels **B**, **D** and **F** show PSGL-1 levels in %, relative to unstimulated control values (media alone), following treatment with inhibitor for 72 h (**B**: **1**; **D**: **2**; **F**: **3**). Data for compound **1** represent mean ± SEM from 11 independent experiments using monocytes isolated from 9 human donors. Data for compound **2** represent mean ± SEM from 7 independent experiments using monocytes isolated from 7 human donors. Data for compound **3** represent mean ± SEM from 9 independent experiments using monocytes isolated from 9 human donors.

In order to obtain a more quantitative measure of its inhibitory activity toward PSGL-1 cell surface presentation, in the presence and absence of IL-1β, we determined full concentration-response curves for compound **1** (1 nM–1 mM) in our PSGL-1 inhibition assay. In the absence of inhibitor, 10 ng/mL IL-1β induced a 3.4-fold increase in PSGL-1 levels compared to unstimulated cells (Fig. 8A). Compound **1** inhibited the IL-1β-induced cell surface presentation of PSGL-1 in a concentration-dependent manner with an IC_50_ of 174 µM (*n* = 5–9, *P* < 0.0001, Fig. 8A), but showed no inhibition of basal PSGL-1 levels (Fig. 8B). Interestingly, however, at sub-maximal concentrations compound **1** did alter basal PSGL-1 levels, with a bell-shaped concentration-response curve having a maximal 3.56 ± 0.69 fold increase at 100 µM (*n* = 5–9, *P* < 0.0001, Fig. 8B) compared to unstimulated monocytes. Monocyte cell viability by trypan blue exclusion was 100% in the presence of 0.5 mM compound **1**.

We also investigated the effect of UDP-GlcNAc derivative **2** and UMP derivative **3** (Fig. 1) on PSGL-1 cell surface presentation under the same conditions. Compound **2** is a congener of compound **1**, possessing a different sugar (**1**: d-galactose; **2**: *N*-acetyl-d-glucosamine), while compound **3** is a possible breakdown product of both **1** and **2**. In the absence of inhibitor, 10 ng/mL IL-1β induced an increase in PSGL-1 levels compared to unstimulated cells, by 3.6-fold (compound **2**, Fig. 8C) and 2.7-fold (compound **2**, Fig. 8E), respectively. Both compounds **2** and **3** showed a similar profile as their parent compound **1**, and reduced IL-1β-induced PSGL-1 levels in a concentration-dependent manner, with an IC_50_ of 77 µM (compound **2**, *n* = 7, *P* < 0.0001, Fig. 8C) and 343 µM (compound **3**, *n* = 9, *P* < 0.0001, Fig. 8E), respectively. While neither compound **2** or **3** inhibited basal PSGL-1 levels, both led to a bell-shaped response, with a maximal increase of 3.5 ± 1.0 fold at 50 µM (compound **2**, *n* = 7, *P* < 0.0001, Fig. 8D) or 2.3 ± 0.3 fold at 100 µM (compound **3**, *n* = 9, *P* < 0.0001, Fig. 8F), compared to unstimulated monocytes. Monocyte cell viability was 100% in the presence of both 0.5 mM compound **2** and 0.5 mM compound **3**.

### Stability tests and cell uptake of inhibitors

**Fig. 9.**
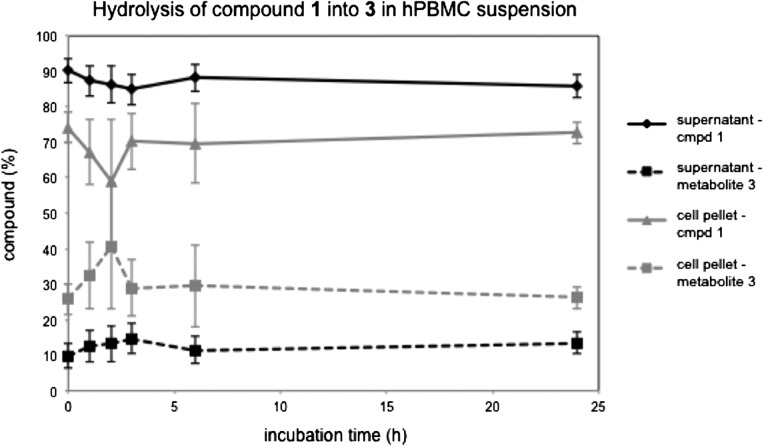
Hydrolysis of 5-FT UDP-Gal (**1**) into 5-FT UMP (**3**) upon incubation with hPBMCs. Cells were incubated with **1** (1 mM) over 24 h and samples taken at different time points. The relative amounts of intact **1** and its metabolite **3** are shown as percentages of the total amount (**1** + **3**) in, respectively, the supernatant (extracellular fraction, from supernatant), or the cell pellet after centrifugation and lysis (intracellular fraction, from cell pellet). Values are mean ± SEM from 4 independent experiments using hPBMCs from 2 human donors.

In order to assess the stability of UDP-sugar **1** and UMP derivative **3** under the conditions of the PSGL-1 inhibition assay, we carried out stability tests in the presence of hPBMCs. In these experiments, compound **1** or **3** (at 1 mM) was incubated for 1–24 h with the hPBMC suspension used in the PSGL-1 inhibition assay. Experiments at each time point were carried out in quadruplicate, with two different batches of hPBMCs. The cell suspension was centrifuged, the supernatant (representing the extracellular fraction of inhibitor) and cell pellet (representing the intracellular fraction of inhibitor) were separated, and the cells of the pellet were lysed with lysis buffer. Both the supernatant and the cell lysate were analyzed by RP-HPLC, using a gradient of 0.05 M phosphate buffer (pH 8) against methanol, which allowed complete separation of compound **3** (retention time: 6.6 min) and compound **1** (retention time: 7.8 min). HPLC traces of the samples incubated with compound **3** showed no other components at UV detection wavelengths of 210, 230, 254 and 280 nm. This result suggests that the UMP derivative **3** is stable in the presence of the hPBMC suspension. In contrast, samples incubated with compound **1** contained between 15% (extracellular fraction) and 30% (intracellular fraction) of **3** over the course of the incubation period (Fig. 9). No other degradation products of compound **1** were observed by HPLC. Maximal levels of the hydrolysis product **3** (extracellular fraction: 15%, intracellular fraction: 30%) were observed already at the first time point, and did not increase significantly over the course of the incubation period. These concentration levels of compound **3** are comparable to those observed in a control experiment at t = 0 h (Fig. 9). This suggests that the observed degradation of compound **1** into compound **3** may happen largely during the work-up step of the stability assay.

**Fig. 10.**
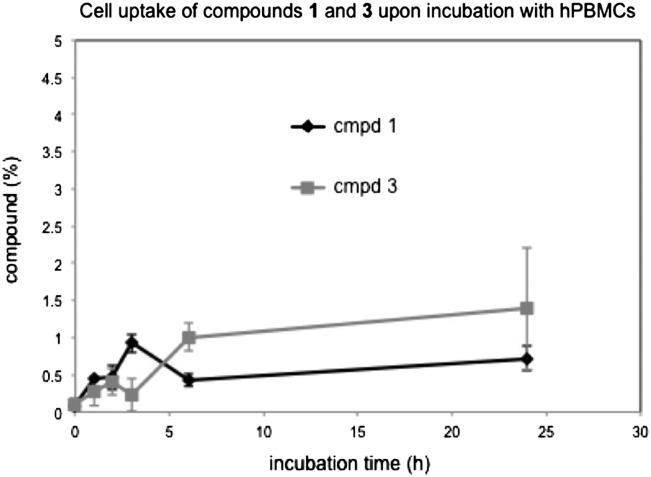
Cellular uptake of 5-FT UDP-Gal (**1**) and 5-FT UMP (**3**) into hPBMCs. Cells were incubated separately with either **1** (1 mM) or **3** (1 mM) over 24 h and samples taken at different time points. The concentrations of **1** and **3** in the cell pellet, after centrifugation and lysis, are shown as percentages of the total concentration used (1 mM). The concentration for 5-FT UDP-Gal **1** is based on the total peak area for both intact **1** and its degradation product **3** (see Fig. 9). Values are mean ± SEM from 4 independent experiments using hPBMCs from 2 human donors.

Finally, we used the results from the stability experiments for a semi-quantitative assessment of the cellular uptake of compounds **1** and **3** by hPBMCs (Fig. 10). From the concentrations of compounds **1** and **3** in the cell pellet, after centrifugation and lysis, we determined the intracellular proportion of compounds **1** and **3** as a percentage of the total concentration used (1 mM). The concentration of 5-FT UDP-Gal **1** in the cell pellet is based on the total peak area for both intact **1** and its degradation product **3** (see Fig. 9). Our HPLC data indicate that approximately 0.5–1% of a 1 mM stock solution of compound **1** are taken up by hPBMCs over 24 h (Fig. 10). Interestingly, the intracellular levels of compound **1** did not change significantly over the course of the incubation period. A similar cell uptake profile was also observed for the UMP derivative **3** (Fig. 10).

## Discussion

In this study, we have determined the constitutive and IL-1β-induced cell surface levels of PSGL-1 on human monocytes by flow cytometry, in order to identify suitable conditions for the evaluation of inhibitors. In IL-1β-stimulated cells, initial PSGL-1 shedding occurred on the cell surface between 1 and 24 hours after IL-1β stimulation, followed by PSGL-1 recovery from 24 to 72 hours. The maximal window between basal and IL-1β-induced PSGL-1 cell surface presentation was observed after 72 h, suggesting that this time point is best suited for the identification and evaluation of chemical inhibitors of IL-1β-induced PSGL-1 cell surface levels. PSGL-1 is highly glycosylated (Wilkins et al. 1996; Somers et al. 2000) and requires the expression of the glycan epitope sLeX for full functional activity (reviewed in Moore 1998). We hypothesized that inhibition of one or more of the GTs required for sLeX biosynthesis (Fig. 1) may reduce the cell surface levels of fully glycosylated PSGL-1. To test this hypothesis, we investigated the effects of the known broad-spectrum GalT inhibitor **1** (Pesnot et al. 2010; Descroix et al. 2012; Tedaldi et al. 2014) as well as the closely related compounds **2** and **3** (Fig. 1) in our assay.

Interestingly, all three test compounds reduced the cell surface levels of PSGL-1 on IL-1β-stimulated hPBMCs, but not on resting cells, as detected by flow cytometry (Fig. 8). The most potent inhibition was observed with the UDP-*N*-acetylglucosamine (UDP-GlcNAc) derivative **2**. There was a significant difference in inhibitory response between compounds **1** and **2** at 100 µM, and between compounds **2** and **3** at 100 µM, 250 µM and 1 mM, (p < 0.05, Fig. 8), while the differences between the inhibitory response of compounds **1** and **3** were not statistically significant. These results indicate that the presence of a sugar (**1**: d-galactose; **2**: *N*-acetyl-d-glucosamine) is beneficial for activity in this class of donor-based GT inhibitors, while the UMP scaffold alone, as in compound **3**, leads to only modest activity. The superiority of the UDP-sugars **1** and **2** over the corresponding UMP derivative **3** is in keeping with well-known structure-activity trends for the inhibition of GTs by sugar-nucleotides and their corresponding mononucleotides. In conjunction with our stability and cell uptake results, the superior activity of compounds **1** and **2** also suggests that it is the complete UDP-sugars, not the potential breakdown product **3**, that are the active agents. However, a more complex mechanism involving the breakdown within cells of **1** and **2** into **3**, followed by re-synthesis to one or more UDP-sugars, cannot be excluded.

At present, our results do not provide a mechanistic explanation for the observed reduction of IL-1β-induced cell surface levels of PSGL-1 by compounds **1**–**3.** We propose a hypothesis based on the observation that GalT inhibitor **1** reduces not only the global presentation of the glycan epitope sLeX on the surface of IL-1β-stimulated hPBMCs (Fig. 5), but also the IL-1β-induced presentation of CD11b, another cell surface glycoprotein decorated with sLeX (Fig. 7). Given the importance of sLeX for PSGL-1 function, this may suggest a link between the effects of compound **1** on PSGL-1 levels on the one hand, and sLeX on the other. Inhibition of sLeX biosynthesis alone cannot, however, account for the observed reduction in IL-1β-induced cell surface levels of PSGL-1 in the presence of compound **1**. Our neuraminidase experiments show, in keeping with previous reports by others (Marathe et al. 2010), that the CD162/KPL-1 antibody used in our assay does not require the presence of sLeX on PSGL-1 for epitope binding. Modifications to the structure of sLeX alone would therefore not be detected by the CD162/KPL-1 antibody. This implies that compound **1** does not just alter the structure of sLeX, on PSGL-1 and other glycoproteins, but genuinely reduces the levels of cell surface PSGL-1 induced by IL-1β. As a possible explanation, we propose that inhibition of sLeX biosynthesis by compound **1** may compromise the trafficking of PSGL-1 to the cell surface. Although a role specifically for sLeX in glycoprotein trafficking has not previously been reported, it is well known that glycosylation affects the intracellular trafficking of glycoproteins and can be manipulated with glycosylation inhibitors (Huet et al. 2003). In preliminary experiments, we have indeed observed that compound **1** does not only reduce IL-1β-induced PSGL-1 levels on the cell surface, but also the intracellular levels of PSGL-1 (unpublished results). These initial findings appear to be consistent with the trafficking hypothesis, but further, quantitative experiments will be required to clarify this.

It is important to note that this mechanism is, at present, hypothetical, and that alternative mechanistic explanations for the observed effects of compounds **1–3** on IL-1β-induced cell surface presentation of PSGL-1 in hPBMCs are conceivable. The lower detection levels of cell surface PSGL-1 by the KPL-1 antibody in the presence of inhibitor may, for example, reflect a more general modification of galactose-containing glycan structures and not just the absence of the sLeX epitope. Davey and coworkers have recently identified galactosyltransferase 4 as a major control point for glycan branching in *N*-linked glycosylation (McDonald et al. 2014). A broad-spectrum GalT inhibitor such as compound **1** (Pesnot et al. 2010) would therefore be expected to substantially alter the structure and composition of eukaryotic *N*-glycans. The inhibition profile of compound **1** toward the four different cell surface glycoproteins investigated in this study narrows down potential intervention points (Table III). Compound **1** is not a general inhibitor of protein *N*-glycosylation in the endoplasmic reticulum, as evidenced by the lack of inhibition toward CD14 and CCR2, which are both *N*-glycosylated (Table III). Neither is compound **1** a general inhibitor of *O*-glycan core structures, as it reduces the cell surface presentation of both PSGL-1, which is *O*-glycosylated, and CD11b, which is not (Table III). Taken together, this profile suggests that compound **1** alters not the core structure, but the antennae and/or terminal epitopes (e.g. sLeX) of affected glycans, possibly by inhibiting one or more GTs resident in the Golgi.

**Table III.** Glycosylation profile of different cell surface glycoproteins, and inhibition of their cell surface levels in hPBMCs by compound **1**

Glycoprotein	Inhibition by cmpd 1	sLeX	*O*-glycans	*N*-glycans
PSGL-1	Yes	Yes (Somers et al. 2000)	Yes (Somers et al. 2000)	Yes (Somers et al. 2000)
CD11b	Yes	Yes (Zen et al. 2007)	No (Sastre et al. 1986)	Yes (Sastre et al. 1986)
CD14	No	*not known*	Yes (Nilsson et al. 2009)	Yes (Meng et al. 2008)
CCR2	No	*not known*	No (Preobrazhensky et al. 2000)	Yes (Preobrazhensky et al. 2000)

Our results in IL-1β-treated hPBMCs are also consistent with a possible effect of our inhibitors on the IL-1 receptor (IL-1R). It is known that glycosylation of IL-1R is required for optimal binding of IL-1 (Mancilla et al. 1992). If the glycan structure on IL-1R was compromised as a result of the activity of inhibitors **1–3**, this may attenuate the stimulation of hPBMCs by IL-1β and result in a reduced presentation of PSGL-1, as observed.

A prerequisite for all three of these hypothetical mechanisms is the cellular uptake of the inhibitors in stable form. In biological media, sugar-nucleotides such as compounds **1** and **2** can be subject to both chemical and enzymatic hydrolysis, due to the presence of several labile bonds, including the glycosidic linkage and the pyrophosphate bond (Huhta et al. 2010). Under the standard hydrolytic pathway for UDP-sugars (Huhta et al. 2010), the primary degradation product of compound **1** would be expected to be UMP derivative **3**. Results from our stability assay indicate that, while compound **3** itself is stable in hPBMC suspension over 24 h, compound **1** is indeed partly hydrolyzed into compound **3**, both intra- and extracellularly (Fig. 9). The unusual time course of degradation suggests, however, that the hydrolysis of compound **1** under the conditions of our stability assay may happen largely during the work-up step. Although it cannot be ruled out completely that UDP-Gal derivative **1** is subject to some hydrolysis during the incubation with hPBMCs, the results from the stability tests indicate that at least 70–85% of intact compound **1** is present under the conditions of the PSGL-1 inhibition assay. This suggests that the observed PSGL-1 inhibitory effect of compound **1** is indeed due to the intact UDP-sugar rather than its breakdown product **3**.

We have previously exploited the fluorescence emission of UDP-sugar **1** to qualitatively investigate its cellular uptake by fluorescence microscopy (Descroix et al. 2012). The quantification of the intra- and extracellular concentrations of compounds **1** and **3** during the stability experiments in the present study has allowed, for the first time, a quantitative assessment of the cellular uptake of compounds **1** and **3** by hPBMCs. Our HPLC data indicate that approximately 0.5–1% of a 1 mM stock solution of compound **1** are taken up by hPBMCs over 24 h (Fig. 10). Interestingly, the intracellular levels of compound **1** did not change significantly over the course of the incubation period. A similar cell uptake profile was also established for the UMP derivative **3** (Fig. 10). Given the charged nature of these molecules, the limited cell penetration of compounds **1** and **3**, even after prolonged incubation, is not surprising. Importantly, although the intracellular concentrations of compound **1** in our experiments are low, they are compatible with the hypothesis that compound **1** reduces cell surface PSGL-1 levels through inhibition of an intracellular GalT, e.g. β-1,4-GalT. In vitro, compound **1** inhibits recombinant β-1,4-GalT with an IC_50_ value of 0.9 μM (Tedaldi et al. 2014). That the IC_50_ value of compound **1** for cellular inhibition of PSGL-1 presentation on hPBMCs (174 μM) is more than 150-fold greater is therefore consistent with its cell uptake being limited to approximately 1%.

While the inhibitory effect of compounds **1–3** on basal cell surface levels of PSGL-1 was minimal, as desired, a modest increase in PSGL-1 presentation was observed in resting cells at submaximal inhibitor concentrations (Fig. 7). This effect disappeared at higher concentrations, leading to a “bell-shaped” concentration-response curve. A possible explanation for this unusual profile may be that in resting cells, low concentrations of compounds **1–3** may be able to induce PSGL-1 presentation, but that at higher concentrations, this effect is off-set by their inhibitory activity. The “bell-shaped” curve may therefore represent the overlay of induction and inhibition of PSGL-1 cell surface levels at different inhibitor concentrations. It is important to note that neither this unusual activity profile in resting hPBMCs, nor the inhibitory effects toward IL-1β-induced PSGL-1 presentation is caused by cytotoxicity, as none of the compounds **1–3** showed any effect on cell viability in the trypan blue assay up to 72 h.

## Conclusion

We have investigated the effect of the GalT inhibitor 5-(5-formylthien-2-yl) UDP-Gal **1**, as well as the corresponding 5-substituted derivatives of UDP-GlcNAc (**2**) and UMP (**3**), on cell surface presentation of PSGL-1 on human monocytes. All three compounds reduce IL-1β-induced cell surface PSGL-1 to basal levels, in a concentration-dependent manner. Compound **1** also inhibits IL-1β-induced presentation of CD11b, but not the cell surface levels of CD14 and CCR2. Together with results from sLeX inhibition experiments, this suggests that these inhibitors act on the cell surface presentation of PSGL-1 via a sLeX-dependent pathway, possibly by affecting glycan-dependent trafficking of PSGL-1 to the cell surface. It is important to note that, on the basis of our current data, this proposed mechanism is hypothetical, and alternative mechanisms of action cannot be ruled out, including e.g. more general modifications of glycan structures or the incorporation of compound **3** into RNA. The potency of compound **1** in inhibiting IL-1β-induced cell surface PSGL-1 levels is limited by its low uptake into cells, and increasing the cell penetration of compound **1** therefore represents an obvious strategy for improving its cellular activity. Intriguingly, under basal conditions, compounds **1–3** have a modest stimulatory effect on PSGL-1 presentation at sub-maximal concentrations. This may indicate that at low concentrations, they may actually induce PSGL-1 presentation in resting cells. While compounds **1–3** themselves may therefore have limited potential for further development, taken together, our results provide proof-of-concept that cell surface levels of PSGL-1 under pro-inflammatory and basal conditions can be differentially modulated with small molecules. These findings therefore open new avenues for intervention of cell surface PSGL-1 levels and may have direct implications for anti-inflammatory drug development.

## Materials and Methods

### Reagents

Compounds **1–3** were prepared as previously described (Pesnot et al. 2010; Tedaldi et al. 2012; Pesnot et al. 2013), purified by ion-exchange and ion-pair chromatography, and used in their triethylammonium salt form. Human recombinant IL-1β was purchased from eBioscience (Hatfield, UK). All antibodies were obtained from BD Biosciences (Oxford, UK) unless otherwise stated. PE-conjugated mouse anti-human CD162 antibody (clone KPL-1, 556055), FITC-conjugated mouse anti-human CD14 antibody (clone M5E2, 555397), PE-conjugated mouse anti-human CD11b/Mac-1 (clone ICRF44, 555388), PE-conjugated mouse IgG2a, K Isotype control antibody (clone G155-178, 555574), purified mouse anti-human CD15s antibody (nonconjugated-anti-CD15s, 551344) and FITC-conjugated goat anti-mouse IgG/IgM antibody (polyclonal, 555988). Alexa Fluor 488 conjugated mouse anti-human CCR2 antibody (clone # 48607, FAB151G) and Alexa Fluor 488 conjugated mouse IgG2B isotype control antibody (clone 133303) were purchased from R&D systems (Abingdon, UK). RPMI 1640 Medium with GlutaMAX™ and Penicillin-Streptomycin (Penstrep) was from Life Technologies Gibco^®^ (Paisley, UK). Bovine serum albumin, phosphate buffered saline, sodium azide, formaldehyde, histopaque-1077 and neuraminidase from *Vibrio cholerae* were from Sigma Aldrich (Dorset, UK).

### Isolation of hPBMCs and treatments.

The study was approved by the national research ethics committee at Guy's and St Thomas’ Hospitals (10/H0807/99). Peripheral venous blood was collected from healthy donors into syringes containing 10% v/v ACD anticoagulant. After complete mixing, blood was added to leucosep^®^ tubes that contained pre-warmed Histopaque-1077 under the barrier. The samples were centrifuged at 1000 g for 10 min. Following centrifugation, mononuclear cells were separated by density from platelets, plasma, granulocytes and red blood cells. Monocyte layers were gently aspirated off and washed twice with media (RPMI-1640 medium with GlutaMAX™ supplemented with 2% FBS, 100 units/mL penicillin and 100 µg/mL streptomycin) in a 5% CO_2_, humidified atmosphere at 37°C. Cell counts were performed, and 0.4 × 10^6^ cells were seeded into each well of a 96-well plate. For IC_50_ experiments, cells were seeded between 1.0 and 2.0 × 10^6^ cells/mL and pre-incubated with media in the presence and absence of compounds (1 nM–1 mM) for 1 h, followed by 10 ng/mL IL-1β in the continued presence of compounds for up to 72 h at 37°C and 5% CO_2_. Cells were harvested and analyzed by flow cytometry. For the 72 h experiments, cell viability to each compound (*n* = 3) was examined using 0.4% Trypan Blue solution. An equal volume of treated cells and Trypan Blue solution was loaded onto haemacytometers, and both viable and non-viable cells were counted. Viability (in %) was calculated as the number of viable cells divided by the total number of cells (both viable and non-viable).

### Flow cytometry

The cell surface levels of PSGL-1 (CD162), sLeX (CD15s), Mac1 (CD11b) and CCR2 were measured by flow cytometry on an Epics XL MCL instrument equipped with an air-cooled laser (488 nm, 15 mW) and a Cytomics FC500 instrument (Beckman Coulter, Florida). For cell surface immunostaining, cells (2 × 10^6^ cells/mL) were centrifuged (1200 rpm, 5 min, 4°C) and resuspended in FACS buffer (PBS supplemented with 1% BSA and 5 mM sodium azide). For neuramidinase experiments, cells were prior fixed with 4% paraformaldehyde. Cells were immunostained for CD162 (1:25), CD15s (1:50), CCR2 (1:25), CD11b (1:25) and their respective isotype controls for 20–30 min at 4°C. FITC/PE-anti-CD14 (1:50) antibodies were used for the measurement of the monocyte population. After incubation, cells were washed twice with FACS buffer and analyzed directly. Samples labeled with sLeX antibody were incubated with a secondary FITC-conjugated Goat Anti-Mouse IgG/IgM antibody, washed again twice with FACS buffer, and resuspended in FACS buffer prior to FACS analysis. 3000 gated cells were analyzed in each experiment with the Coulter System II software, and the percentage and the mean fluorescence intensity (MFI) were used to quantify the responses. For Cytomics FC500, CXP software was used to quantify responses.

### Statistical analysis

All raw data (either MFI or % of cells) are presented as mean ± SEM. Fold change data were collected from individual donor treatments by normalizing to respective unstimulated controls and presented as mean ± SEM experiments. Data for dose- and time-dependency of PSGL-1 levels upon incubation of hPBMCs with IL-1β for 0–72 h, including the effect of inhibitor **1** at 1 mM, were analyzed with one-way analysis of variance (ANOVA), followed by Bonferroni's multiple-comparisons test. Data for the effect of compound **1** on PSGL-1 or sLeX levels at 72 h were analyzed using two-way analysis of variance (ANOVA), followed by Bonferroni post-test. All analyses were performed with GraphPad Prism and IC_50_ curves for normalized data were generated by a four-parameter sigmoidal model (version 5.0; GraphPad, San Diego, CA). A *P* value less than 0.05 was considered signiﬁcant.

### Stability tests

#### Incubation with inhibitors

On a 96-well microplate, 200 µL of hPBMC suspension at 2 × 10^6^ cell/mL was added per well. Inhibitors **1** or **3** (final concentration: 1 mM) were added to individual wells and incubated for 1, 2, 3, 6 and 24 hours. Samples were collected and immediately centrifuged at 1300 x *g* for 5 minutes. The supernatant (representing the extracellular inhibitor fraction) was separated, freeze-dried and stored at −80°C until quantification. To the pellet (representing the intracellular inhibitor fraction), 185 µL of cold 0.5 M PCA was added. The sample was mixed, incubated on ice for 2 minutes and centrifuged at 10,000 x *g* for 5 minutes. After centrifugation, the supernatant was transferred to a new vial, and 42 µL of cold 2.5 M KOH in 1.5 M K_2_HPO_4_ was added. The solution was incubated on ice for 2 minutes and centrifuged at 10,000 x *g* for 5 minutes. The supernatant was filtered through a 2 µm filter, and the filter membrane was washed twice with 100 µL of ultra pure H_2_O. The filtrate was freeze-dried and stored at −80°C until quantification. Each inhibitor concentration was tested in quadruplicate.

#### Quantification of inhibitor fractions

All samples (cell pellet or supernatant) were analyzed by reverse-phase high performance liquid chromatography (RP-HPLC) on a Perkin Elmer 200 machine equipped with a Supelcosil LC-18-T column (5 µm, 25 cm × 4.6 mm), an autosampler and a diode array detector. Detection wavelengths: 254, 210, 230 and 280 nm. Reference wavelength: 360 nm. Flow rate: 1.5 mL/min. Gradient: 0.05 M phosphate buffer (pH 8) against methanol. Solvents were purchased from Fisher Scientific and were of HPLC-grade quality. Extracellular samples were dissolved in 90 µL of H_2_O (injection volume: 30 µL). Intracellular samples were dissolved in 90 µL of H_2_O (24 h, injection volume: 30 µL), 60 µL of H_2_O (6 h, injection volume: 60 µL), or 30 µL of H_2_O (1, 2 and 3 h, injection volume: 30 µL). The smaller total volume was required for the intracellular samples because of the relatively low concentration of inhibitor. Retention times: **1** – 7.8 min, **3** – 6.6 min.

#### Data analysis

Samples were quantified based on Total Peak Area (TPA) in the respective HPLC chromatogram. In the presence of hPBMCs, compound **1** is hydrolyzed into compound **3** both intra- and extracellularly. To assess the stability of compound **1**, the percentage of intact **1** relative to the combined amount of compound **1** and degradation product **3** was determined at a given time point, in both the cell pellet and supernatant. To assess the cell uptake of compound **1**, the percentage of the combined amounts of intact compound **1** and degradation product **3**, relative to the total amount of compound **1** used (1 mM), was determined in the cell pellet at a given time point.

## Funding

This work was supported by a grant from the King's Health Partners R&D Challenge Fund (grant number R120523), with additional support from the Medical Research Council (grants number G0701861 and G0901746, to G.K.W.). JJ is the recipient of a King's College London PGR studentship.

## Abbreviations

CD14, cluster of differentiation 14; CD162, cluster of differentiation 162; FACS, fluorescence-activated cell sorting; FBS, fetal bovine serum; FITC, fluorescein isothiocyanate; 5-FT UDP-Gal, 5-(5-formylthien-2-yl) uridine diphosphate α-d-galactose; GT, glycosyltransferase; GalT, galactosyltransferase; hPBMC, human peripheral blood mononuclear cells; IL-1β, interleukin-1β; MFI, mean fluorescence intensity; PCA, perchloric acid; PE, phycoerythrin; PSGL-1, P-selectin glycoprotein-1; RP-HPLC, reverse phase high performance liquid chromatography; sLeX, Sialyl Lewis X; TPA, total peak area; UDP-Gal, uridine diphosphate α-d-galactose; UDP-GlcNAc, uridine diphosphate α-d-*N*-acetylglucosamine; UMP, uridine monophosphate
